# Influence of Health Insurance Types on Clinical Cancer Care Accessibility and Quality Using *All of Us* Database

**DOI:** 10.3390/medicina60040623

**Published:** 2024-04-11

**Authors:** Sedra Tibi, Vivian Tieu, Suat Babayigit, Jun Ling

**Affiliations:** Department of Medical Education, School of Medicine, California University of Science and Medicine, Colton, CA 92324, USA; sedra.tibi@md.cusm.edu (S.T.); vivian.tieu@md.cusm.edu (V.T.); suat.babayigit@cusm.edu (S.B.)

**Keywords:** socioeconomic factors, insurance, health inequity, care accessibility, cancer

## Abstract

*Background and Objectives*: Cancer, as the second leading cause of death in the United States, poses a huge healthcare burden. Barriers to access to advanced therapies influence the outcome of cancer treatment. In this study, we examined whether insurance types affect the quality of cancer clinical care. *Materials and Methods*: Data for 13,340 cancer patients with Purchased or Medicaid insurance from the *All of Us* database were collected for this study. The chi-squared test of proportions was employed to determine the significance of patient cohort characteristics and the accessibility of healthcare services between the Purchased and Medicaid insurance groups. *Results*: Cancer patients who are African American, with lower socioeconomic status, or with lower educational attainment are more likely to be insured by Medicaid. An analysis of the survey questions demonstrated the relationship between income and education level and insurance type, as Medicaid cancer patients were less likely to receive primary care and specialist physician access and more likely to request lower-cost medications. *Conclusions*: The inequities of the US healthcare system are observed for cancer patient care; access to physicians and medications is highly varied and dependent on insurance types. Socioeconomic factors further influence insurance types, generating a significant impact on the overall clinical care quality for cancer patients that eventually determines treatment outcomes and the quality of life.

## 1. Introduction

In 2021, the CDC reported that the three leading causes of death in the United States were heart disease, cancer, and COVID-19 [1]. Neoplastic cells have the ability to evade the human immune system, making cancers very difficult to diagnose and treat. According to the American Cancer Society, there were 1.9 million new cancer cases and a projected 609,360 deaths from cancers in 2022 [2].

In the United States of America (USA), health insurance and coverage influence the outcomes of healthcare, including diagnosis, prognosis, and the quality of life for patients [3,4,5]. Medicaid is a US federal government program that provides affordable, low-cost, or free healthcare to low-income American individuals and families, those with disabilities, pregnant women, and the elderly [6]. Patients with low socioeconomic status (SES) are typically eligible for Medicaid, whereas those who do not qualify may turn to Purchased insurance instead (e.g., Aetna, Blue Cross Blue Shield Association, Kaiser Permanente, etc.). Medicaid expansion (MES) due to the Affordable Care Act has reduced racial disparities in healthcare and improved cancer diagnosis and treatment [7]. Specifically, MES resulted in statistically significant decreases in chemotherapy delays for African American and Hispanic breast cancer patients and decreased advanced stages of disease at diagnosis for rural breast cancer patients [7,8]. Increased screening and cancer detection, and decreased mortality were also observed for Stage II and III rectal cancer patients covered by Medicaid [9].

Although Medicaid is historically for underserved populations; recent studies have revealed disparities and gaps for cancer patients at “high-quality care” institutions [10]. Limited numbers of participating institutions, physicians, and specialists are amongst many reasons as to why Medicaid coverage does not guarantee equal access to quality care and may lead to fragmented care, delayed care, and inaccessibility to more costly immunotherapeutics for cancer patients [11,12]. Such factors must be considered to understand the disparities in cancer survival for Medicaid patients [13].

With the expanding Medicaid patient population, it is crucial to address the lack of universal and standardized care for cancer patients. The goal of this study is to examine whether insurance status (Medicaid versus Purchased) influences the accessibility and quality of clinical care for cancer patients using the *All of Us* (*AoU*) database. By analyzing the factors that influence insurance status, we hope to identify the roles sociodemographics play in impacting cancer patients’ access to quality clinical care and to raise awareness of the inequities in the American healthcare system.

## 2. Materials and Methods

### 2.1. Data Source

Data were obtained from the *AoU* Research Program database, which employs Observational Medical Outcomes Partnership (OMOP) Common Data Model Version 5 infrastructure to compile and standardize data from electronic health records (EHRs) for researchers. Enrollment for *AoU* began in May 2018 and contains data for those ≥18 years old from more than 340 recruitment sites in the USA. Information was obtained from EHRs, health questionnaires, physical measurements, the use of digital health technology such as Fitbit data, and the collection and analysis of biospecimens. With more than 175,000 participants, the variety of socioeconomic, lifestyle, and biologic characteristics represents populations in the United States. To ensure proper data security and organization, The Data and Research Center in Nashville, TN, USA houses the database information. Funded by the National Institute of Research (NIH), the *AoU* Research Program aims to deliver large and thorough datasets to advance medical research. The *AoU* dataset consists of EHR data from various OMOP sources and data domains including Demographics, Conditions, Procedures, Drugs, Measurements, and Visits [14].

### 2.2. Cohort Selection

**Figure 1:**
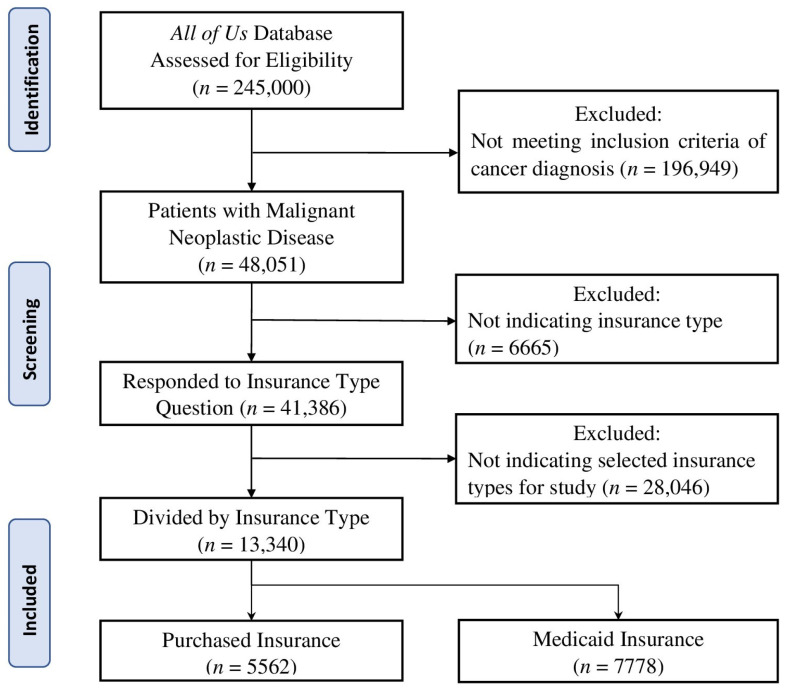
CONSORT flow diagram for selection of participants. A diagram to convey the screening and selection process of participants chosen for analysis in this study.

We used the *AoU* database to identify patients ≥18 years old, diagnosed with malignant neoplastic diseases, and indicated their insurance type as Purchased or Medicaid. Data were obtained from version 7 of the *AoU* database, which includes participant data from the start of enrollment in May 2018 until July 2022. Participant selection can be viewed in Figure 1. Data for this project were extracted in June 2023. Of the 245,000 participants assessed for eligibility in the *All of Us* database, 48,051 met criteria for diagnosis of malignant neoplastic disease. From this participant sample, 41,386 indicated their insurance type and 13,340 had the two insurances of interest for this study: 5562 Purchased insurance holders and 7778 Medicaid insurance holders. The screening and selection of participants for this study is conveyed in Figure 1.

### 2.3. Outcomes Variables 

Covariate analysis was focused on sociodemographic characteristics including age, sex at birth (male, female, or not answered), race (classified as Asian, Black/AA, White, None Indicated, or None of These), annual household income, and educational attainment level. The term “Black/AA” was used for consistency with *AoU* database categorization. Patients were classified by insurance coverage type (Purchased or Medicaid). 

### 2.4. Statistical Analysis 

Sociodemographic information was summarized using descriptive statistics. The chi-squared test of proportions was used to analyze the significance of patient cohort characteristics (age, sex at birth, race, annual household income, and educational attainment level) between Purchased and Medicaid insurance groups. Survey questions were employed to analyze the proportions of patients who indicated that their health insurance was not accepted by a healthcare provider or office, could not afford to see a specialist or primary care physician when needed, or had requested lower-cost medications. The chi-squared test was used to evaluate the association between categorical variables, and all statistical tests were two-tailed, and *p*-values < 0.05 were considered statistically significant. All statistical tests were performed using SPSS 28 (IBM Corp. Released 2021. IBM SPSS Statistics for Windows, Version 28.0. Armonk, NY, USA).

## 3. Results

### 3.1. Patient Cohort Characteristics and Insurance Groups

We extracted data from 13,340 patients with malignant neoplastic diseases either on Purchased (5562) or Medicaid (7778) insurance from the *All of Us* database. A chi-squared test for independence was performed to examine the relationship between insurance types and patient cohort characteristics. The results indicated that there was a statistically significant association between insurance type and age, with individuals aged 65+ years old more likely to have Purchased insurance (*n* = 4618, 83.0%) compared to Medicaid (*n* = 3068, 39.4%), $X^{2}=2544.09,\;p<0.001$. A greater proportion of females held Purchased insurance (*n* = 3100, 56.2%) compared to males (*n* = 2416, 43.8%), $X^{2}=111.10,\;p<0.001$. White individuals were more likely to have Purchased insurance (*n* = 4723, 92.9%) compared to other races, $X^{2}=2053.34,\;p<0.001$. The highest proportion of Medicaid holders had annual incomes less than USD 25 K (*n* = 4118, 76.6%), whereas the majority with other incomes between USD 50 and USD 100 K had Purchased insurance (*n* = 1526, 33.6%), $X^{2}=4699.45,\;p<0.001$. Those with a college degree or higher were more likely to have Purchased insurance (*n* = 3227, 58.7%) compared to those with less education, $X^{2}=2655.54,\;p<0.001$.

We observed a predominance of patients aged 65 years or older in the Purchased insurance group (*n* = 4618, 83.0%) and a relatively equal distribution of 45–64-year-old (44.9%) and 65+-year-old (39.4%) patients in the Medicaid group. Compared to the Purchased group, the Medicaid group had a predominance of females to males of 2:1. We found that the patients in the Purchased insurance group were more likely to identify their race as White compared to the Medicaid insurance group (92.9% vs. 54.1%, *p* < 0.001). The patients in the Medicaid group were more likely to identify their race as African American compared to the Purchased insurance group (44.1% vs. 5.6%, *p* < 0.001). The patients in the Medicaid insurance group specified their annual household income predominantly in the USD <25 K group compared to the patients in the Purchased insurance group (76.6% vs. 11.5%, *p* < 0.001). When comparing the levels of educational attainment, most of the patients (58.7%) in the Purchased insurance group described themselves as having a college graduate or advanced degree, while the patients in the Medicaid insurance group disclosed <12th grade (20.2%), 12th grade or GED (29.7%), or college education (31.1%). Essentially, the Medicaid insurance group had a predominance of women, individuals of African American race, a household income of USD < 25 K annually, and a college or below education level in comparison to the Purchased insurance group (Table 1).

### 3.2. Healthcare Access and Insurance Types

**Figure 2:**
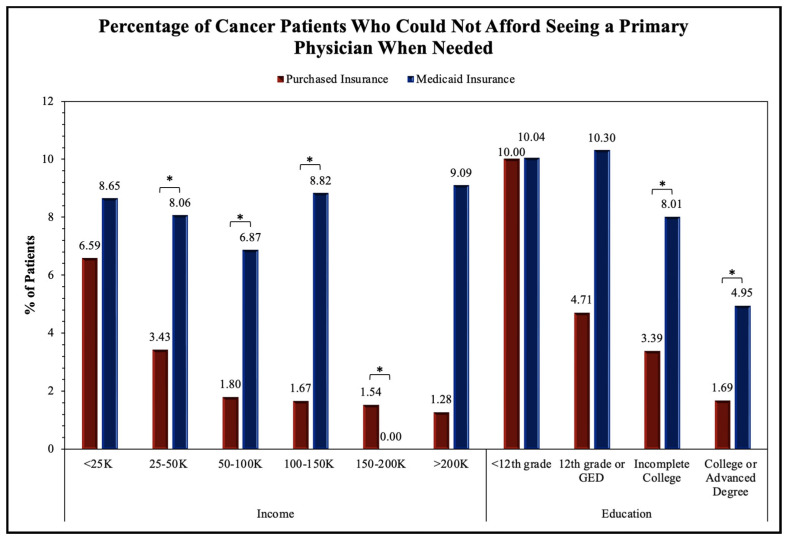
Impact of insurance type on cancer patient ability to see primary care physician in relation to income and education. Proportion of cancer patients who indicated they could not afford to see a primary care physician when needed in the last twelve months was sorted by annual household income and the level of highest educational attainment. Frequencies were statistically compared by insurance types of Purchased and Medicaid in each income and education category, with an asterisk (*) indicating statistical significance within each group.

**Figure 3:**
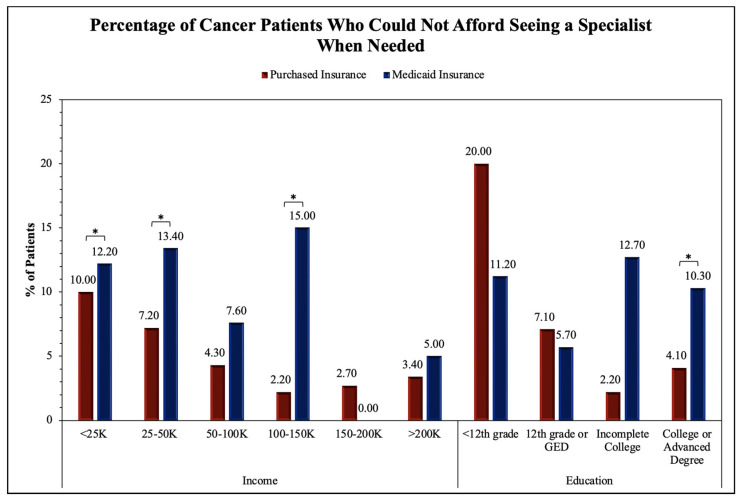
Impact of insurance type on cancer patient ability to see specialists in relation to income and education. Proportion of cancer patients who indicated they could not afford to see a specialist physician when needed in the last twelve months was sorted by annual household income and the level of highest educational attainment. Frequencies were statistically compared by insurance types of Purchased and Medicaid in each income and education category, with an asterisk (*) indicating statistical significance within each group.

**Figure 4:**
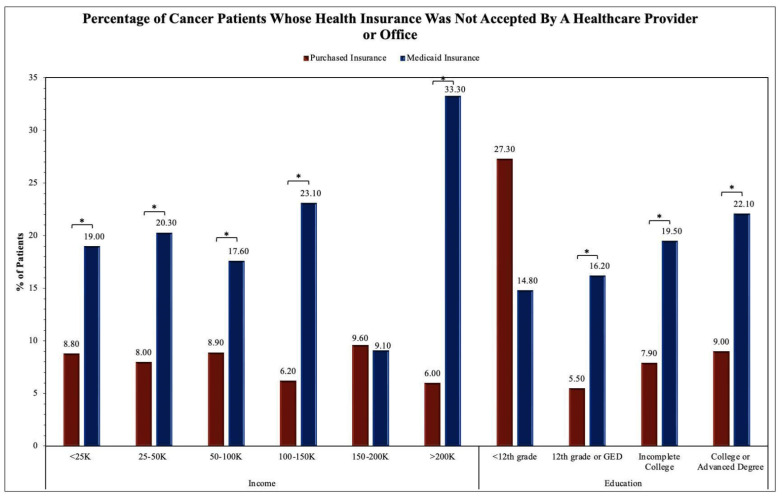
Insurance type determines rates of insurance acceptance for cancer patients in relation to income and education. Proportion of cancer patients who indicated their health insurance was not accepted by a healthcare provider or office in the last twelve months was sorted by annual income and the level of highest educational attainment. Frequencies were statistically compared by insurance types of Purchased and Medicaid in each income and education category, with an asterisk (*) indicating statistical significance within each group.

**Figure 5:**
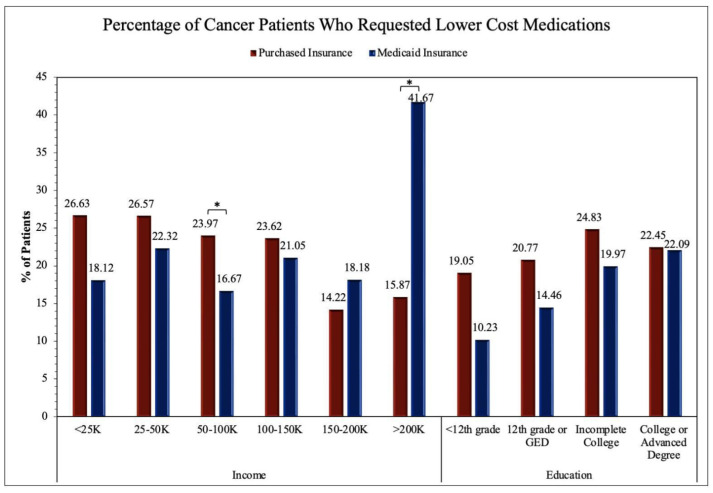
Impact of insurance type on cancer patient rates of requesting lower-cost medications in relation to income and education. Proportion of cancer patients who requested lower-cost medications in the last twelve months sorted by annual income levels and the highest educational attainment. Frequencies were statistically compared by insurance types Purchased and Medicaid in each income and education category, with an asterisk (*) indicating statistical significance.

To assess the accessibility to healthcare for patients with malignant neoplastic diseases, we analyzed many relevant questions in the survey using the chi-squared test; thus, the statistical significance between the Purchased and Medicaid insurances was revealed. Regarding the general accessibility to healthcare services, there was statistical significance in the proportion of patients whose health insurance was not accepted at a healthcare office (*p* < 0.001), were unable to afford co-pay (*p* < 0.001), and were unable to receive follow-up care due to not being able to afford it (*p* < 0.001). No significance was found for patients having a high/unaffordable deductible (*p* = 0.299) and paying out of pocket for a procedure (*p* = 0.639). The survey questions pertaining to access to primary and specialist care showed a statistically significant difference between Purchased and Medicaid insurances for being unable to see a primary care physician (*p* < 0.001) or a specialist (*p* < 0.001) due to financial reasons in the past twelve months. No significance was found for having seen a primary care physician (*p* = 0.364) or a specialist (*p* = 0.066) in the past twelve months. The evaluation of the access to therapeutics showed a statistical significance between the Purchased and Medicaid groups for skipping medication doses to save money (*p* < 0.001), asking for a lower-cost medication to save money (*p* < 0.001), delaying filling a prescription to save money (*p* < 0.001), and being unable to obtain a prescription medication due to being unable to afford it (*p* < 0.001). All these results are shown in Table 2 and Figure 2, Figure 3, Figure 4 and Figure 5.

The upcoming figures (Figure 2, Figure 3, Figure 4 and Figure 5) will further analyze prominent healthcare services by comparing the service accessibility between the Purchased and Medicaid insurances in relation to income and educational levels. The participants from the *All of Us* database selected the annual household income level that best described their household, with six options (USD <25 K, USD 25–50 K, USD 50–100 K, USD 100–150 K, USD 150–200 K, and USD >200 K). Similarly, the participants selected their highest educational attainment with four categories (<12th grade, 12th grade or GED, incomplete college, and college or advanced degree). In this study, these two factors (annual household income and educational level) are reflections of financial status and resource accessibility.

Figure 2 analyzes the proportion of cancer patients unable to see their primary care physician. The two-proportion Z-test was applied to obtain results for comparing the Purchased and Medicaid category frequencies. Significance between the Purchased and Medicaid insurance groups was found in the USD <25 K (Z-score = −3.32, *p* < 0.001), USD 25–50 K (Z-score = −2.87, *p* = 0.004), and USD 100–150 K (Z-score = −4.05, *p* < 0.001) income levels in addition to the college or advanced degree education levels (Z-score = 3.51, *p* < 0.001). The remainder of the income levels, including USD 50–100 K, USD 150–200 K, and USD >200 K, and the education levels of <12th grade, 12th grade or GED, and those with an incomplete college education were not found to show significant differences between the Purchased and Medicaid insurance groups.

Figure 3 displays the analysis of the proportion of cancer patients unable to see a specialist. The two-proportion Z-test was applied to obtain results for comparing the Purchased and Medicaid category frequencies. Significance between the Purchased and Medicaid insurance groups was found in the USD 25–50 K (Z-score = −2.88, *p* = 0.004), USD 50–100 K (Z-score = −3.46, *p* = 0.001), USD 100–150 K (Z-score = −2.73, *p* = 0.006), and USD >200 K (Z-score = −2.00, *p* = 0.046) income levels in addition to the incomplete college (Z-score = −3.79, *p* < 0.001) and college or advanced degree education levels (Z-score = −4.34, *p* < 0.001). The remainder of the income levels, including USD <25 K and USD 150–200 K and education levels <12th grade and 12th grade or GED were not found to show significant differences between the Purchased and Medicaid insurance groups.

The two-proportion Z-test was applied to obtain results for comparing the Purchased and Medicaid category frequencies in Figure 4. Significance between the Purchased and Medicaid insurance groups was found in the USD 50–100 K (Z-score = 2.53, *p* = 0.012) and USD >200 K (Z-score = −3.24, *p* = 0.001) income levels. The remainder of the income levels, including USD <25 K, USD 25–50 K, USD 100–150 K, and USD 150–200 K and all education levels including <12th grade, 12th grade or GED, incomplete college, and college or advanced degree education were not found to show significant differences between the Purchased and Medicaid insurance groups.

The two-proportion Z-test was applied to obtain results for comparing the Purchased and Medicaid category frequencies in Figure 5. Significance between the Purchased and Medicaid insurance groups was found in the USD <25 K (Z-score = −3.73, *p* < 0.001), USD 25–50 K (Z-score = −5.49, *p* < 0.001), USD 50–100 K (Z-score = −3.29, *p* < 0.001), USD 100–150 K (Z-score = −3.88, *p* < 0.001), and USD >200 K (Z-score = −3.60, *p* < 0.001) income levels in addition to the education levels of 12th grade or GED (Z-score = −4.65, *p* < 0.001), incomplete college (Z-score = −6.82, *p* < 0.001), and college or advanced degree education (Z-score = −8.88, *p* < 0.001). The remaining income level of USD 150–200 K and education level of <12th grade were not found to show significant differences between the Purchased and Medicaid insurance groups.

## 4. Discussion

In our analysis, we identified multiple SES factors associated with differences in insurance types. The younger (<64 years old) and female patients were more likely than the older (65+ years old) and male patients to have Medicaid insurance. We observed that the White patients were more likely than the Black/AA patients to have Purchased insurance. Overall, the patients with lower household incomes and levels of educational attainment were more likely to have Medicaid insurance than Purchased insurance. As prognosis and outcomes for malignant conditions vary widely between insurance types, it can be assumed that those who are Black/AA, have a lower SES status, or have lower educational attainment, are more likely to have a poorer cancer prognosis and quality of life. The survey questionnaire responses from the Purchased and Medicaid insurance holders helped establish the difference in the accessibility of healthcare services. Highly related survey questions were chosen and analyzed by annual household incomes and educational levels. We found that fundamental healthcare services such as seeing a primary care physician (Figure 2) and insurance acceptance (Figure 4) demonstrated significant differences between Purchased and Medicaid insurances compared to specialized healthcare services such as seeing a specialist (Figure 3) or requesting lower-cost medication (Figure 5). Those with lower education levels and Purchased insurance tend to have higher rates of difficulty when attempting to see a primary care physician, affording a specialist, and seeking lower cost medications compared to their Medicaid counterparts (Figure 2, Figure 3, Figure 4 and Figure 5).

As seen in our findings, those with Medicaid insurance had similar difficulty across income groups regarding insurance being accepted by a healthcare provider (Figure 4). This suggests that Medicaid insurance may offer consistent care accessibility within its insurance group and is independent of income factors. In contrast, the findings in the Purchased insurance group suggest income and educational levels play a large role in determining accessibility and the high variability of ease of accessibility within Purchased insurance. For this reason, future research should be directed toward analyzing each insurance program independently rather than grouping them together, which may misrepresent and generalize findings to all Purchased insurance companies. However, it is important to address that several income and education levels throughout the four survey questions (Figure 2, Figure 3, Figure 4 and Figure 5) did not demonstrate the same statistically significant difference, indicating variability of accessibility within both Purchased and Medicaid insurances. As cancer is a high-mortality and -morbidity disease, patients have access to specialist care and standard chemotherapeutics regardless of insurance type or SES status, which may explain the results seen in Figure 4 and Figure 5 [15].

The results of our study are consistent with previous literature, as historically, African American and other minority groups have had lower rates of private insurance compared to their White counterparts [16]. Inadequate healthcare insurance has been shown to impact cancer patient survival. Previous studies have also established that African American patients have the lowest survival rate and highest mortality of any racial group for most cancers, including colorectal cancer [17] and HCC [18,19]. One study reported uninsured African American patients with metastatic colorectal cancer had lower rates of receipt of chemotherapy and higher mortality rates compared with White patients and those with private insurance [20]. Moreover, a 2020 retrospective study demonstrated how Medicaid and uninsured cancer patients did not receive additional survival benefits from experimental therapies compared with their private insurance counterparts [21]. As an individual’s healthcare insurance is largely determined by their SES, it is safe to say that an individual’s SES can dictate the quality of healthcare one receives.

Our analysis findings are consistent with previous literature, as a recent national survey showed that although 99% of physicians were accepting new patients, 25.5% did not accept Medicaid insurance [22]. In addition, Medicaid cancer patients were more likely to report that they did not receive care due to cost, delayed care due to cost, or did not take prescription drugs due to cost compared with privately insured cancer patients [22]. A 2020 retrospective study also concluded that Medicaid or uninsured breast, colon, and lung cancer patients failed to receive recommended chemotherapy [23]. Such variation in cancer care delivery and accessibility is consistent with our study’s findings. Moreover, such shortcomings in Medicaid insurance aid in explaining how Medicaid cancer patients are more likely to exhibit poorer disease outcomes. Published studies support this hypothesis, as late-stage overall survival in young cancer patients was significantly longer in those with Purchased insurances than those with public insurances [4]. A 2022 meta-analysis found that those with Medicaid and uninsured patients were more likely to be diagnosed in the late stages (stage III/IV) of cancer and had worse short-term and long-term survival compared to those with Purchased insurance [14]. Furthermore, there was higher mortality risk amongst breast cancer patients with Medicaid insurance compared to those with private insurance [24]. Notably, a 2020 study showcased no significant difference in survival between small cell lung cancer patients who had Medicaid and those who were uninsured [25]. The findings in our analysis are consistent with the literature analyzed above, which utilized multivariable logistic regressions with similar datasets as those in our study.

Although insurance plays a significant role in the quality and accessibility of medical services, it is also important to address the institutional, societal, racial, and cultural factors that contribute to the healthcare discrepancies in the United States. Despite the 2010 Affordable Care Act’s best efforts in extending health insurance coverage to many more qualifying American citizens, it is still insufficient in combating the health inequities and disparities faced by underserved communities [26]. Moreover, Hao et al. showcased that regardless of insurance type, African American and Hispanic patients are still less likely to receive standard care for cancer [27]. Barriers to receiving and accepting standard cancer care may extend beyond insurance type, including medical mistrust, fatalism and negative surgical beliefs amongst African American patients [28,29]. Overall, our study revealed how societal and economic categories have overarching consequences related to healthcare, provider, and therapeutic access that may drastically impact a cancer patient’s prognosis and survival likelihood.

One of the limitations of our study is that our data were limited to the *AoU* database, which contains missing data that could further shed light on the differences between insurance groups. Information regarding cancer-free states, survival, and overall prognosis was missing from the available data, preventing comparative analyses on these factors. Another limitation is the structure of the dataset. The socioeconomic information listed in Table 1 was not associated with the individual level, thereby preventing us from performing multivariate logistic regression on the survey questions.

## 5. Conclusions

This study utilizes the very broad *All of Us* database to examine the socioeconomic determinants of health faced by cancer patients, how those factors are correlated with insurance types and healthcare access. Our results identified the persistent socioeconomic and racial disparities in the American healthcare system.

By highlighting the shortcomings and inequities of the American health system, we hope that improvements in these areas can be made, and future health policies can be implemented to not limit patients’ healthcare access based on their SES, race, or education level. As cancer is a life-threatening disease with very limited therapeutic options, it is crucial for every cancer patient to receive equal, prompt, and high-quality care in order to improve the overall disease outcomes.

## Figures and Tables

**Table 1:** Comparison of demographic characteristics by insurance groups in the sampled cohort. Distribution of the proportions of participants in each category were compared between Purchased and Medicaid insurance to analyze statistical differences. All categories were indicated in the table.

	Characteristic	Purchased Insurance Holder	Medicaid Insurance Holder	χ^2^, *p*
	Total number of subjects	5562	7778	
Age, n (%)	18–44 years old	156 (2.8)	1221 (15.7)	2544.09, <0.001
	45–64 years old	788 (14.2)	3489 (44.9)	
	65+ years old	4618 (83.0)	3068 (39.4)	
Sex at Birth, n (%)	Male	2416 (43.8)	2667 (34.7)	111.48, <0.001
	Female	3100 (56.2)	5012 (65.3)	
Race, n (%)	Asian	76 (1.5)	94 (1.8)	2053.34, <0.001
	Black or African American	285 (5.6)	2326 (44.1)	
	White	4723 (92.9)	2858 (54.1)	
Annual Household Income, n (%)	USD <25 K	521 (11.5)	4118 (76.6)	4699.45, <0.001
	USD 25–50 K	1076 (23.7)	836 (15.5)	
	USD 50–100 K	1526 (33.6)	321 (6.0)	
	USD 100–150 K	694 (15.3)	63 (1.2)	
	USD 150–200 K	299 (6.6)	18 (0.3)	
	USD >200 K	421 (9.3)	23 (0.4)	
Educational Attainment, n (%)	<12th grade	103 (1.9)	1530 (20.2)	2655.54, <0.001
	12th grade or GED	736 (13.4)	2250 (29.7)	
	College	1434 (26.1)	2354 (31.1)	
	College graduate or advanced degree	3227 (58.7)	1444 (19.1)	

**Table 2:** Comparison of survey questions evaluating accessibility to healthcare services between the two insurance types. Proportions of participants who indicated “Yes” or “No” to encountering the stated situation were statistically compared using chi-squared analysis between Purchased and Medicaid insurances to evaluate the significance. All questions refer to patient experiences within the past twelve months.

	Survey Question	Survey Response	Purchased Insurance Holder (*n* = 4676)	Medicaid Insurance Holder (*n* = 6663)	Chi-square, *p*-Value
Access to General Healthcare	Were you told by a health care provider or doctor’s office that they did not accept your health care coverage?	Yes, n (%)	217 (8.0)	347 (19.5)	127.13, <0.001
		No, n (%)	2480 (92.0)	1430 (80.5)	
	Were you delayed in receiving care for any of the following reasons due to not affording the co-pay?	Yes, n (%)	86 (3.9)	132 (8.4)	33.53, <0.001
		No, n (%)	2092 (96.1)	1431 (91.6)	
	Were you delayed in receiving care due to a high or unaffordable deductible?	Yes, n (%)	122 (5.6)	100 (6.5)	1.078, 0.299
		No, n (%)	2041 (94.4)	1448 (93.5)	
	Were you delayed in receiving care due to being required to pay out of pocket for some or all of the procedure?	Yes, n (%)	274 (12.7)	206 (13.2)	0.220, 0.639
		No, n (%)	1885 (87.3)	1353 (86.8)	
	Was there a time when you needed follow-up care but could not receive it due to being unable to afford it?	Yes, n (%)	81 (3.7)	153 (9.7)	55.71, <0.001
		No, n (%)	2087 (96.3)	1421 (90.3)	
Access to Primary Care	Have you seen or spoken about your own health with a general doctor/primary care provider?	Yes, n (%)	2490 (93.9)	1629 (93.2)	0.823, 0.364
		No, n (%)	161 (6.1)	118 (6.8)	
	Was there a time when you needed to see a general care provider but could not due to being unable to afford it?	Yes, n (%)	56 (2.5)	126 (7.8)	58.14, <0.001
		No, n (%)	2164 (97.5)	1482 (92.2)	
Access to Specialist Care	Have you seen or spoken about your own health with a specialist (other than a general provider, obstetrician/gynecologist, psychiatrist, or ophthalmologist)?	Yes, n (%)	1660 (80.7)	1131 (78.2)	3.38, 0.066
		No, n (%)	397 (19.3)	316 (21.8)	
	Was there a time when you needed to see a specialist but could not due to being unable to afford it?	Yes, n (%)	104 (4.7)	191 (12.0)	67.93, <0.001
		No, n (%)	2090 (95.3)	1398 (88.0)	
Access to Therapeutics	Have you skipped medication doses to save money?	Yes, n (%)	123 (4.6)	186 (10.4)	56.64, <0.001
		No, n (%)	2568 (95.4)	1605 (89.6)	
	Have you asked your doctor for a lower cost medication to save money?	Yes, n (%)	584 (23.9)	309 (18.2)	19.61, <0.001
		No, n (%)	1855 (76.1)	1390 (81.8)	
	Have you delayed filling a prescription to save money?	Yes, n (%)	198 (7.7)	233 (13.4)	36.70, <0.001
		No, n (%)	2367 (92.3)	1511 (86.6)	
	Was there a time when you needed to obtain a prescription medication but could not due to being unable to afford it?	Yes, n (%)	212 (8.0)	363 (20.6)	1150.19, <0.001
		No, n (%)	2452 (92.0)	1398 (79.4)	

## Data Availability

Data used in this study are available as a featured workspace to registered researchers of the *All of Us* Researcher Workbench. For information about access, please visit https://www.researchallofus.org/.
